# Beyond the Device: Why Provider-Side Factors Determine Upper-Limb Prosthesis Acceptance

**DOI:** 10.7759/cureus.112966

**Published:** 2026-07-19

**Authors:** Mitsunori Toda

**Affiliations:** 1 Orthopaedic Surgery/Rehabilitation Medicine, Hyogo Prefectural Rehabilitation Central Hospital, Kobe, JPN

**Keywords:** clinician learned non-engagement, learned non-use, motor learning, neuroplasticity, prosthesis abandonment, prosthesis embodiment, prosthetic rehabilitation, shared decision-making, upper-limb amputation, upper limb prosthesis

## Abstract

Abandonment of upper-limb prostheses has remained stubbornly common for more than two decades, despite substantial refinement of prosthetic hardware. A recent survey from a leading bionic-reconstruction group found that technological advancement did not significantly change acceptance rates; instead, structured prosthetic training and active patient participation in decision-making emerged as the factors most likely to improve device use. Read together with earlier work identifying fitting time-frame and client involvement as critical variables, these data relocate the problem of abandonment from the device to the process of rehabilitation. This editorial argues that the decisive variables in prosthesis acceptance are provider-side: the timing, structure, and shared-decision quality of the rehabilitation a patient receives. Drawing on the concept of learned non-use from stroke rehabilitation, I propose-as an interpretive framework rather than a demonstrated mechanism-that clinicians themselves may develop a parallel clinician learned non-engagement (LNE), a conditioned reduction of active engagement shaped by the same behavioral contingencies that drive patient non-use. If the field's central obstacle is no longer the hardware, then attention, measurement, and resources should shift toward the human processes that determine whether a well-engineered hand is ever truly adopted. Practically, this argues for building rehabilitation timing and provider engagement into care pathways as routinely measured outcomes, and for prioritizing prospective study of these process variables.

## Editorial

A persistent paradox

For more than 25 years, the rehabilitation community has confronted an uncomfortable fact: a large proportion of people fitted with an upper-limb prosthesis eventually stop using it. A comprehensive survey of the preceding quarter-century documented rejection and abandonment rates for active prostheses that frequently approached or exceeded half of all users [1]. The intuitive explanation has always been technological. If devices were heavier, slower, and less capable than the biological hand they replaced, then better devices (lighter, more dexterous, more intuitively controlled) should close the gap and abandonment should fall.

That intuition has now been tested directly. Salminger and colleagues surveyed traumatic upper-limb amputees and grouped them by the era of their amputation to ask whether a decade of prosthetic innovation had moved the needle on acceptance. It had not: there was no significant difference in acceptance between patients amputated before or after 2006, and the overall rejection rate remained at 44% [2]. The weight of this finding is amplified by its source. It comes from a group at the forefront of bionic extremity reconstruction: precisely the people whose work defines the technological frontier. When the builders of better hands report that better hands did not, by themselves, change adoption, the field is obliged to ask a different question: if not the device, then what?

Locating the variable on the provider side

The same survey that dismissed technology pointed toward an answer. Its authors concluded that well-structured, patient-tailored prosthetic training, together with genuine patient participation in the decision-making process, would most likely improve prosthetic acceptance [2]. A small but telling observation reinforced the point: the respondents who had received no prosthetic training at all were non-users. The sample is too small to prove anything on its own, but the direction is consistent and worth stating plainly.

This conclusion does not stand alone. In a separate analysis of the critical factors distinguishing users from non-users, Biddiss and Chau found that among enabling resources, it was the fitting time-frame and the involvement of the client in prosthesis selection that weighed on the decision to abandon a device [3]. Two independent lines of evidence therefore converge on the same territory, not the mechanical properties of the prosthesis, but the process of care surrounding it: how early the team acts, how the training is structured, and whether the patient is treated as a participant rather than a recipient. These are not properties of the hardware. They are properties of the rehabilitation the health system chooses to deliver.

A framework for why: learned non-use, extended

Why should the process of care matter so much more than the device? Stroke rehabilitation offers a well-developed framework. The phenomenon of learned non-use describes how, after neurological injury, a limb with sufficient residual capacity is nonetheless left unused, not because movement is impossible, but because early failure and the reinforced success of compensatory strategies condition the patient to suppress its use. As Taub and colleagues articulated, this is a behavioral outcome driven by altered contingencies of reinforcement, producing motor disability in excess of what the injury alone would warrant [4]. The essential insight is that non-use is learned, and therefore, in principle, unlearnable.

The parallel to the abandonment of prostheses is direct. A new prosthesis user who struggles with an unfamiliar, effortful device while discovering that one-handed compensation is faster and more reliable is being conditioned, trial by trial, toward non-use of the prosthesis. Abandonment, on this reading, is not a verdict on the device so much as the predictable product of a contingency structure that rewards giving up. This is why training and early success matter: they alter the contingencies before non-use is consolidated.

There is a less comfortable extension of this logic, which I offer as an interpretive hypothesis rather than a demonstrated mechanism. The definition of learned non-use is not intrinsically restricted to patients. Its core, that a capable agent reduces an action because the surrounding contingencies fail to reinforce it, describes a general behavioral pattern. If that is so, the clinical team is not exempt. When active engagement with prosthetic rehabilitation is effortful, poorly reimbursed, squeezed by competing acute demands, or unsupported by the systems around it, clinicians may drift toward a parallel state that I will call clinician learned non-engagement (LNE): a conditioned reduction of active engagement with the prosthetic rehabilitation process, shaped by the same behavioral logic that produces non-use in patients. (I use engagement here to denote the provider's active involvement in the rehabilitation process, distinct from the more familiar sense of patient engagement.) Where the patient learns not to use the device, the provider may learn not to invest in the process that makes the device usable. The reimbursement structure makes this concrete: in many systems, funding covers the device itself but rarely the clinical hours that structured training requires, so the work most likely to secure adoption is the work least likely to be paid for. Framed this way, reduced engagement is less a personal failing than a predictable response to the incentives the system supplies, which is why I treat LNE as a systemic and economic constraint as much as a behavioral one. I emphasize that this is not an accusation of individual negligence; like patient non-use, it names a pattern produced by contingencies, and precisely because it is learned, it is something a system can be designed to prevent.

LNE remains a conceptual proposal, not an established finding: testing it would require longitudinal observation of provider behavior and the contingencies that shape it, which does not yet exist. But naming it has value even before it is proven, because it reframes what the convergent data already show. If provider-side factors determine acceptance, and if provider behavior is itself governed by contingencies, then the recurrent under-delivery of structured, timely, participatory rehabilitation is not simply a matter of individual diligence. It is a systemic pattern that behaves like learned non-use, and, like learned non-use, it should be addressable by redesigning the contingencies rather than by exhorting individuals to try harder. That is the practical promise of the framework: a problem that is learned is a problem that can be unlearned by design.

Engagement has a window

If provider engagement is decisive, then its timing is not a detail but a determinant. Four decades ago, Malone and colleagues, reviewing immediate, early, and late prosthetic fitting after upper-limb amputation, described what they termed a “Golden Period” for fitting within roughly the first month after amputation, and recommended fitting as rapidly as possible [5]. The clinical stakes of that window are considerable. In that early series, patients fitted within the first month returned to work at rates approaching the entire group, whereas those fitted later did so at only a small fraction, a gradient later echoed by repeated associations between early fitting and more successful long-term prosthesis use. The figures come from a small, historical cohort and should not be read as precise probabilities; but the direction and the magnitude are difficult to ignore.

Framed through the present argument, the Golden Period is best understood not as a target for the rehabilitation physician to hit, but as a property of the system that delivers care. A missed window is rarely the patient's failure, and it is seldom the failure of any single clinician's diligence; more often it reflects delays in decision-making, referral, funding approval, and socket provision (processes distributed across institutions and largely outside the control of the rehabilitation team that eventually receives the patient). In many settings, including my own, the referral for prosthetic rehabilitation does not even arrive until well after the first month has passed. This is precisely LNE expressed at the level of the system rather than the individual: the contingencies that link surgical care to rehabilitation are structured in a way that quietly trains timely engagement out of the pathway. Administrative delay in device approval is one such contingency, converting a solvable scheduling problem into a permanent abandonment.

If that is the correct reading, then capitalizing on the Golden Period cannot be achieved by rehabilitation providers working harder within their own walls, because the decisive delays occur upstream, before the patient is ever referred. The realistic first step is structural: early information-sharing between the surgical teams that perform amputations and the rehabilitation services that will fit and train the prosthesis, ideally beginning at the surgical planning stage, well before discharge. Such a channel need not be elaborate; a shared expectation that rehabilitation is engaged early, as a default rather than an afterthought, would itself redesign the contingency. This is the sense in which a learned pattern can be unlearned by design: not by exhorting individuals, but by building the connective structures that make timely engagement the path of least resistance for everyone involved. Advocating for that connection (patiently, and across the institutional boundary between surgery and rehabilitation) may be among the most consequential things those of us in rehabilitation can do.

Where the field should look

The convergent message of these studies is not that prosthetic engineering is unimportant; it is that engineering is no longer the binding constraint on adoption. The lever now lies in the rehabilitation process: in how early we fit, how deliberately we structure training, and how genuinely we share the decision with the person who will live with the device. These process variables deserve the same seriousness we extend to hardware: they should be built into care pathways, measured routinely, and reported as outcomes in their own right. Realizing this will often require reaching across the institutional boundary between surgery and rehabilitation, so that engagement begins early enough to matter.

None of this is to suggest that provider-side process is the only thing that matters. Acceptance is also shaped by socioeconomic circumstance, device cost, the adequacy of reimbursement, and the patient’s own psychological adaptation to limb loss, which frequently unfolds as a grieving process in which the early introduction of a demanding device can itself provoke rejection; on this view, early engagement may sometimes mean attending first to readiness and coping rather than to fitting. The timing argument has boundaries as well: where the elective, pre-surgical planning I describe is impossible, as after traumatic or emergency amputation, the same principle has to be pursued through the earliest feasible postoperative contact rather than before surgery. I bracket these factors here not because they are minor, but because this editorial’s focus is deliberately on the provider-side processes that clinicians and systems can most directly redesign.

Conclusions

Better hands will keep arriving. Whether patients keep them depends less on the hands than on us: on whether the systems we work within reinforce timely, structured, participatory engagement, or quietly train it out of us. Confronting the possibility of clinician learned non-engagement is uncomfortable, but it is also hopeful: contingencies that produce non-use can be redesigned to produce use. A fuller account of how motor-learning and embodiment principles can guide that redesign is a task for a subsequent, more comprehensive treatment. Testing these ideas will require concrete work: prospective studies that measure provider engagement directly, trials of standardized early-referral and rehabilitation pathways, and interventions designed to sustain clinician participation over time. The first and most immediate step, however, is a change of focus: to look beyond the device, and toward the human processes that decide whether it is ever truly adopted.
